# Brain structural and functional substrates of *ADGRL3* (*latrophilin 3*) haplotype in attention-deficit/hyperactivity disorder

**DOI:** 10.1038/s41598-021-81915-z

**Published:** 2021-01-27

**Authors:** Ana Moreno-Alcázar, Josep A. Ramos-Quiroga, Marta Ribases, Cristina Sánchez-Mora, Gloria Palomar, Rosa Bosch, Josep Salavert, Lydia Fortea, Gemma C. Monté-Rubio, Erick J. Canales-Rodríguez, Michael P. Milham, F. Xavier Castellanos, Miquel Casas, Edith Pomarol-Clotet, Joaquim Radua

**Affiliations:** 1 FIDMAG Research Foundation, C/. Dr. Antoni Pujadas, 38, Sant Boi de Llobregat, 08830 Barcelona, Spain; 2 Centre Forum Research Unit, Institute of Neuropsychiatry and Addictions (INAD), Hospital del Mar, Barcelona, Spain; 3 Institut Hospital del Mar d’Investigacions Mèdiques, Barcelona, Spain; 4 Biomedical Network Research Centre on Mental Health (CIBERSAM), Instituto de Salud Carlos III, Madrid, Spain; 5 Department of Psychiatry and Legal Medicine, Universitat Autònoma de Barcelona, Barcelona, Spain; 6 Psychiatric Genetics Unit, Group of Psychiatry, Mental Health and Addiction, Vall d’Hebron Research Institute (VHIR), Vall d’Hebron Hospital Universitari, Vall d’Hebron Barcelona Hospital Campus, Universitat Autònoma de Barcelona, Barcelona, Spain; 7 Servei de Psiquiatria, Vall d’Hebron Hospital Universitari, Vall D’Hebron Barcelona Hospital Campus, Barcelona, Spain; 8 Department of Genetics, Microbiology & Statistics, University of Barcelona, Barcelona, Spain; 9 Department of Psychiatry, Sant Rafael Hospital, Barcelona, Spain; 10 Institut d’Investigacions Biomèdiques August Pi i Sunyer (IDIBAPS), C/. Rosselló, 149, 08036 Barcelona, Spain; 11 Institute of Neurosciences, University of Barcelona, Barcelona, Spain; 12 Nathan Kline Institute for Psychiatric Research, Orangeburg, NY USA; 13 Center for the Developing Brain, Child Mind Institute, New York, NY USA; 14 Department of Child and Adolescent Psychiatry, Hassenfeld Children’s Hospital at NYU Langone, New York, NY USA; 15 Signal Processing Lab (LTS5), École Polytechnique Fédérale de Lausanne (EPFL), Lausanne, Switzerland; 16 Centre for Psychiatric Research and Education, Department of Clinical Neuroscience, Karolinska Institutet, Stockholm, Sweden; 17 Department of Psychosis Studies, Institute of Psychiatry, Psychology and Neuroscience, King’s College London, London, UK

**Keywords:** Functional magnetic resonance imaging, Magnetic resonance imaging, Genotyping and haplotyping, ADHD

## Abstract

Previous studies have shown that the gene encoding the adhesion G protein-coupled receptor L3 (*ADGRL3*; formerly *latrophilin 3, LPHN3*) is associated with Attention-Deficit/Hyperactivity Disorder (ADHD). Conversely, no studies have investigated the anatomical or functional brain substrates of *ADGRL3* risk variants. We examined here whether individuals with different *ADGRL3* haplotypes, including both patients with ADHD and healthy controls, showed differences in brain anatomy and function. We recruited and genotyped adult patients with combined type ADHD and healthy controls to achieve a sample balanced for age, sex, premorbid IQ, and three *ADGRL3* haplotype groups (risk, protective, and others). The final sample (n = 128) underwent structural and functional brain imaging (voxel-based morphometry and n-back working memory fMRI). We analyzed the brain structural and functional effects of ADHD, haplotypes, and their interaction, covarying for age, sex, and medication. Individuals (patients or controls) with the protective haplotype showed strong, widespread hypo-activation in the frontal cortex extending to inferior temporal and fusiform gyri. Individuals (patients or controls) with the risk haplotype also showed hypo-activation, more focused in the right temporal cortex. Patients showed parietal hyper-activation. Disorder-haplotype interactions, as well as structural findings, were not statistically significant. To sum up, both protective and risk *ADGRL3* haplotypes are associated with substantial brain hypo-activation during working memory tasks, stressing this gene’s relevance in cognitive brain function. Conversely, we did not find brain effects of the interactions between adult ADHD and *ADGRL3* haplotypes.

## Introduction

Attention-deficit/hyperactivity disorder (ADHD) is one of the most frequent behavioral psychiatric disorders in childhood; it affects ~ 5–6% of children and adolescents and has impairing symptoms that persist in more than 50% of adults^1^. The disorder is characterized by pervasive inattention and/or hyperactivity and impulsivity, associated with social and/or educational/occupational impairments^2^. Some patients have inattention predominantly (e.g., they make careless mistakes, are forgetful, etcetera). Other patients have hyperactivity and impulsivity mainly (e.g., they get up often when seated, talk out of turn, etcetera). Still, most patients have a “combined type” ADHD involving inattentive and hyperactive-impulsive symptoms.

Previous studies have reported several environmental factors that may increase the risk of ADHD, such as maternal pre-pregnancy overweight, preeclampsia, hypertensive disorders, acetaminophen exposure or smoking during pregnancy, childhood eczema or asthma, or vitamin D deficiency^3^. However, family, twin, and adoption studies have repeatedly demonstrated the substantial influence of genetic factors, with heritability estimated to be around 76%^4,5^.

Meta-analyses of candidate genes and genome-wide association studies (GWAS) have identified several genes and loci associated with ADHD^6–9^. One leading candidate gene is the *BAIAP2* (brain-specific angiogenesis inhibitor 1-associated protein 2), which has shown a consistent association with ADHD across studies^8^ and meta-analytic statistical significance even after Bonferroni correction. Besides, a recent meta-analysis has highlighted another candidate gene repetitively associated with ADHD, the *ADGRL3* (adhesion G protein-coupled receptor L3; formerly *LPHN3*, latrophilin 3)^9–12^.

Magnetic resonance imaging (MRI) studies have described several structural and functional brain abnormalities in patients with ADHD, including decreased gray matter volume in the motor area, prefrontal cortex, and age-dependent volume in basal ganglia^13–16^. Studies have also identified reduced brain response to cognitive tasks in the same brain regions, although the evidence is still weak and needs further investigation^15–20^.

Given the association between *ADGRL3* haplotype and ADHD, and the consistency of structural and functional brain studies, we aimed to investigate the relationship between *ADGRL3* haplotypes and the brain abnormalities to characterize the brain structural and functional differences between ADHD patients and controls depending on their *ADGRL3* haplotype. To this end, we recruited, genotyped, and MRI-scanned 64 patients with combined type ADHD and 64 healthy controls, balanced for age, sex, premorbid IQ, and *ADGRL3* haplotypes. We exploratorily hypothesized that beyond the brain structural and functional effects of ADHD, there could be effects of the haplotypes and even effects of the interaction between ADHD and the haplotypes. The latter would mean that the brain correlates of ADHD depend on the haplotype. This finding would be interesting to understand the disease better and pave the way for a haplotype-based personalization of non-invasive brain stimulation therapy^21^.

## Results

### Participants

The final sample included 128 participants (64 patients and 64 controls), of which 42 (21 patients and 21 controls) were homo- or heterozygous for the protective haplotype, and 51 (28 patients and 23 controls) were homo- or heterozygous for the risk haplotype. As shown in Table 1, there were no substantial differences between patients and controls or between haplotype groups on age (mean: patients 36, controls 36, protective haplotype 36, risk haplotype 37, and other haplotypes 35 years; SD around 12 years), sex (patients 66%, controls 48%, protective haplotype 57%, risk haplotype 61%, other haplotypes 51% males), and premorbid IQ (TAP score mean: patients 23, controls 23, protective haplotype 23, risk haplotype 23, other haplotypes 23; SD around 5). The ratio of homozygosis vs. heterozygosis of the risk haplotype was higher in patients with ADHD than in healthy controls (patients 14 homozygous and 14 heterozygous, healthy controls: 2 homozygous and 21 heterozygous, chi-square p-value = 0.004).

**Table 1:** Description of the sample.

	All participants	ADHD status		Haplotype		
		Patients	Healthy controls	Protective	Risk	Other
**All haplotypes**						
Group size	128	64	64	42	51	35
Age (mean ± SD)	36.2 ± 11.9	36.2 ± 12.0	36.2 ± 11.8	36.0 ± 10.8	37.1 ± 12.3	35.3 ± 12.6
Sex (% males)	57.0%	65.6%	48.4%	57.1%	60.8%	51.4%
TAP score (mean ± SD)	22.9 ± 4.9	22.6 ± 5.1	23.1 ± 4.7	22.9 ± 5.0	23.0 ± 5.0	22.7 ± 4.7
Homo/heterozygosis	–	–	–	9/33	16/35	–

TAP: “*Test de Acentuación de Palabras*” (an indicator of premorbid IQ).

### Structural findings

We did not observe any statistically significant ADHD or *ADGRL3* haplotype effects after correction for multiple comparisons.

**Figure 1:**
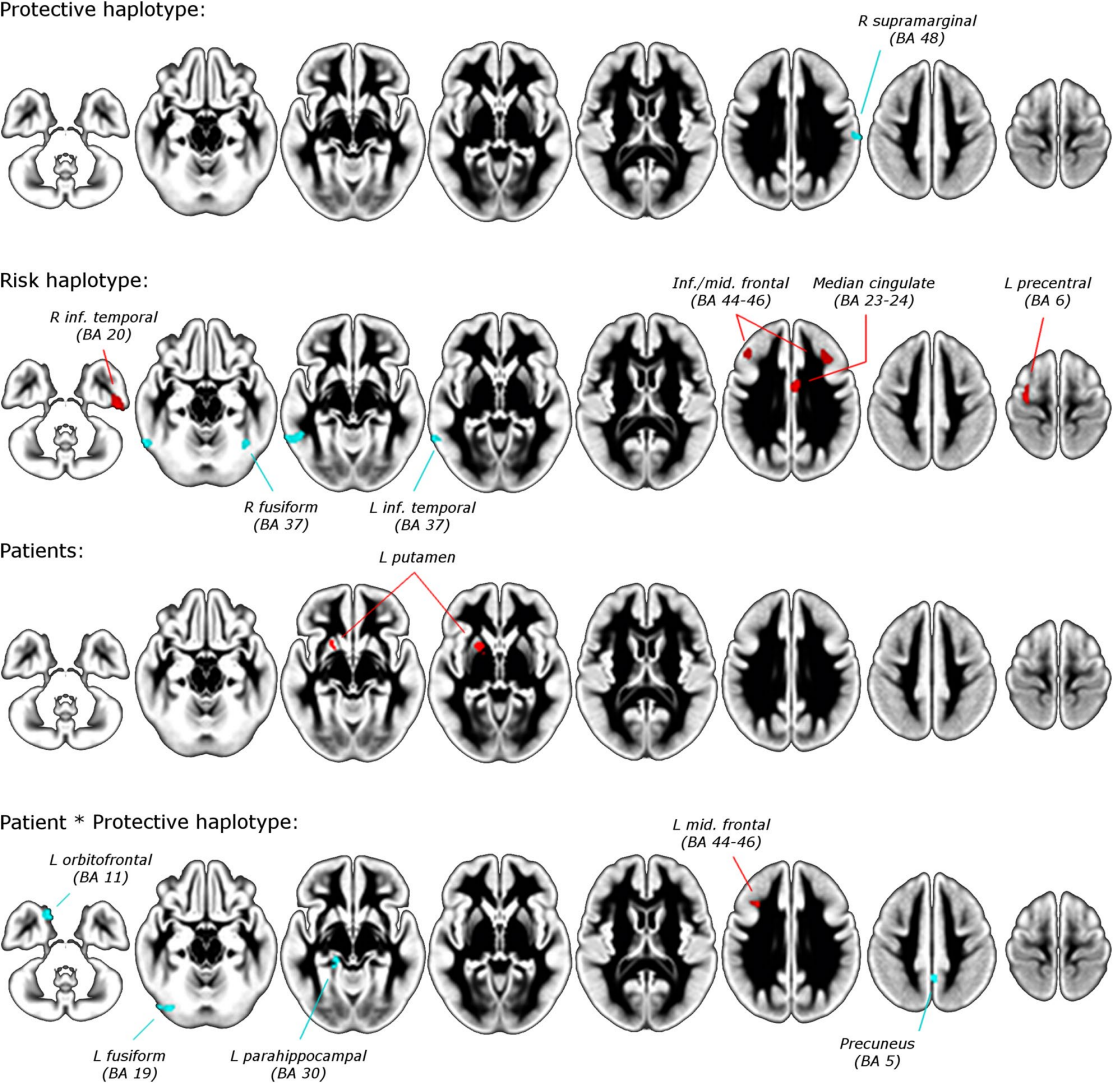
Brain increases and decreases of gray matter volume depending on *ADGRL3* haplotypes and the attention-deficit/hyperactivity disorder (ADHD) status, using uncorrected p-value < 0.001. Top: Volumetric differences in individuals (patients or controls) with the protective haplotype. Middle/top: Volumetric differences in individuals (patients or controls) with the risk haplotype. Middle/bottom: Volumetric differences in patients with ADHD relative to healthy subjects. Bottom: Volumetric interaction between ADHD status and protective haplotype. Areas in red indicate significantly more gray matter in individuals with the haplotype or patients or a positive interaction between the disorder and the haplotype; blue areas indicate substantially less gray matter in individuals with the haplotype or negative interaction between the disorder and the haplotype. Differences were not statistically significant after correction for multiple comparisons.

However, for the sake of exhaustivity, we report here (but not further discuss) the findings at uncorrected p < 0.001 level (Fig. 1 and Table 2). First, individuals (patients or controls) with the protective haplotype showed decreased gray matter in the right supramarginal gyrus compared to individuals without the protective haplotype (see details in Table 2). Second, individuals (patients or controls) with the risk haplotype showed increased gray matter in several frontotemporal regions and decreased gray matter in inferior temporal/fusiform gyrus compared to individuals without the risk haplotype. Third, patients with ADHD showed increased gray matter in the left putamen as compared to healthy controls. Finally, we observed interactions between the protective haplotype and ADHD status in the left middle frontal gyrus, orbitofrontal, fusiform, parahippocampal gyri, and precuneus. The interaction in the left middle frontal gyrus was positive: we observed a trend-level increase in individuals with protective haplotype, a trend-level increase in patients, and an extra increase in patients with protective haplotype. The interactions in the other regions were negative: we did not observe any overall effect of protective haplotype or disorder, but a decrease in patients with protective haplotype.

**Table 2:** Effects of ADHD and *ADGRL3* haplotypes on gray matter volume (Contrasts Patients < controls, Protective > other haplotypes, and Patients with Risk > or < Controls with other haplotypes, did not returned significant results).

		Peak MNI	Peak t-value	Cluster size	Cluster breakdown
**Gray matter decreases in individuals with protective haplotype**^**a,b**^					
Modulated	R supramarginal	60, -20, 24	4.116	116	R supramarginal (112)
**Gray matter increases in individuals with risk haplotype**^**a,b**^					
Unmodulated	Median cingulate	4, − 2, 32	4.285	99	Median cingulate (67)
	L precentral	− 30, − 14, 58	4.331	81	L precentral (72)
Modulated	R inf. temporal	52, − 18, − 38	4.668	197	R inf. temporal (123)
	R mid. frontal	38, 24, 30	3.563	76	R mid. frontal (15)
	L inf. frontal	− 42, 30, 28	3.734	72	L inf. frontal (33) L mid. frontal (29)
**Gray matter decreases in individuals with risk haplotype**^**a,b**^					
Modulated	L inf. temporal	− 56, − 52, − 12	4.004	269	L inf. temporal (165) L mid. temporal (89)
	R fusiform	40, − 58, − 20	3.549	100	R fusiform (54) R cerebellum (39)
**Gray matter increases in patients**					
Unmodulated:	L putamen	− 18, 8, − 4	3.613	61	L putamen (13)
Modulated:	L putamen	− 18, 6, − 2	3.678	53	L putamen (10)
**Interaction: gray matter increases in patients with protective haplotype**^**b,c**^					
Unmodulated	L mid. frontal	− 32, 22, 32	4.545	50	L mid. frontal (9)
**Interaction: gray matter decreases in patients with protective haplotype**^**b,d**^					
Unmodulated	L orbitofrontal	− 16, 16, − 24	3.787	98	L orbitofrontal (29) L sup. temporal (22) L mid. temporal (16)
	L fusiform	− 34, − 78, − 16	3.956	71	L fusiform (39)
	L parahippocampal	− 18, − 32, − 10	3.920	54	L parahippocampal (3)
Modulated	Precuneus	2, − 50, 50	3.478	90	Precuneus (90)

*Inf.* Inferior, *L* left, *mid.* Middle, *R* right, *sup.* superior.

^a^In the absence of disorder-haplotype interactions.

^b^We compared risk and protective haplotype to other haplotypes.

^c^Trend-level increase in individuals with protective haplotype, trend-level increase in patients, and extra increase in patients with protective haplotype.

^d^No overall effects of protective haplotype or disorder, but decrease in patients with protective haplotype.

### Functional findings

The performance of the different groups in the n-back task was mostly similar. We only observed a marginal increase of d’ in individuals with the protective haplotype in the 2-back task (t = 2.04, uncorrected p-value = 0.043).

**Figure 2:**
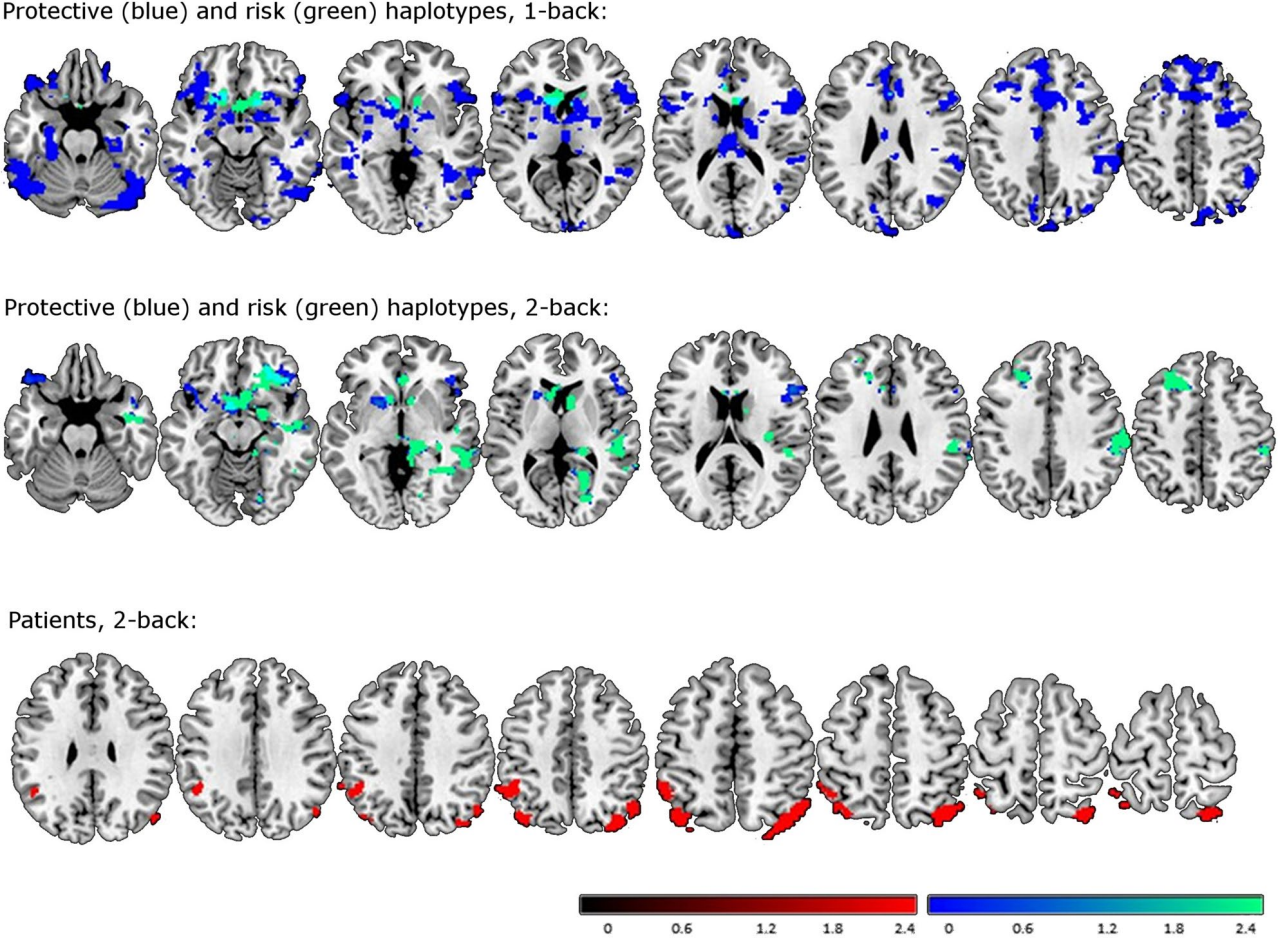
Brain hyper- and hypo-activation depending on *ADGRL3* haplotypes and the attention-deficit/hyperactivity disorder (ADHD) status during the N-back task. Top: Hypo-activation in individuals (patients or controls) with the protective haplotype (blue) and with the risk haplotype (green) during the performance of the 1-back task vs. baseline. The light-blue color shows overlap regions in both groups. Middle: Hypo-activation in individuals (patients or controls) with the protective haplotype (blue) and with the risk haplotype (green) during the performance of the 2-back task vs. baseline. The light-blue color shows overlap regions in both groups. Bottom: Hyper-activation in patients with ADHD relative to healthy control during the 2-back vs. baseline task. Color bars indicate z-values from the group-level analysis.

The fMRI study’s main finding was a widespread hypo-activation in the 1-back vs. baseline contrast in individuals (patients or controls) with the protective haplotype (compared to individuals without the protective haplotype; Table 3 and Fig. 2). This hypo-activation especially included the inferior, superior, and middle frontal gyri, the inferior and middle temporal gyri, the anterior and median cingulate cortices, and to a lesser extent, the fusiform gyrus, the cuneus/precuneus, the supplementary motor area, the inferior and middle occipital gyri, the cerebellum, the supramarginal gyrus, the insula, the thalamus, the caudate, the parahippocampal gyrus, the putamen, and several other regions (see details in Table 3). The 2-back vs. baseline comparison showed hypo-activation, although this was less statistically significant and more circumscribed to the frontal gyri.

**Table 3:** Effects of ADHD and *ADGRL3* haplotypes on the brain response during n-back task (Contrasts Patients < controls, Risk > other haplotypes, and interactions, did not returned significant results).

		Peak MNI	Peak z-value	Cluster size	Cluster p-value	Cluster breakdown
**Hypo-activation in individuals with protective haplotype**^**a,b**^						
1-back:	Frontal/cingulate	− 4, − 20, 18	5.03	10,064	< 0.001	L sup. frontal (1011) L inf. frontal (798) R sup. frontal (599) L mid. frontal (556) Ant. cingulate (547) Median cingulate (519) R mid. frontal (459) L supplementary motor area (294) R supplementary motor area (281) L insula (248) R caudate (133) R precentral (128) L putamen (124) L thalamus (100) R thalamus (97) L caudate (61) R putamen (58) R insula (41) L sup. temporal (39) R inf. frontal (27) L gyrus rectus (16)
	R temporal/fusiform	14, − 88, − 18	4.24	4382	< 0.001	R inf. temporal (526) R fusiform (381) R mid. temporal (356) R cerebellum (320) Cuneus (255) Precuneus (223) R mid. occipital (149) R parahippocampal (129) R inf. occipital (127) R lingual (89) R sup. occipital (87) R angular (87) R sup. parietal (70) L calcarine (44) L sup. occipital (38) R calcarine (31) L sup. parietal (11)
	R inf. frontal	58, 16, 14	4.47	1356	< 0.001	R inf. frontal (1100) R insula (21) R sup. temporal (13) R Rolandic operculum (12) R precentral (10)
	L inf. temporal	− 52, − 46, − 18	4.53	901	0.007	L inf. temporal (507) L fusiform (153) L cerebellum (111) L mid. temporal (24) L inf. occipital (19)
	L fusiform	− 26, − 32, − 20	3.86	871	0.009	L fusiform (164) L mid. temporal (75) L parahippocampal (65) L hippocampus (61) L inf. temporal (18) L insula (13) L cerebellum (13) L putamen (10)
	R supramarginal	60, − 38, 34	4.28	853	0.011	R supramarginal (386) R inf. parietal (179) R sup. temporal (62)
2-back:	R inf. frontal	52, 50, 16	3.87	843	0.008	R inf. frontal (567) R mid. frontal (52) R sup. frontal (39) R sup. temporal (39)
	L inf. frontal	− 8, 2, − 10	3.83	710	0.021	L inf. frontal (150) L putamen (45) L insula (27) L sup. temporal (12)
**Hypo-activation in individuals with risk haplotype**^**a,b**^						
1-back:	L caudate	− 6, 18, 6	3.87	1.063	0.002	L caudate (115) Ant. cingulate (54) L olfactory (38) R caudate (34) R olfactory (29) R gyrus rectus (22) L gyrus rectus (18)
2-back:	R temporal/supramarginal	52, − 32, 6	4.11	3881	< 0.001	R mid. temporal (408) R supramarginal (303) R sup. temporal (231) Ant. cingulate (124) R inf. frontal (99) R calcarine (83) R caudate (76) L caudate (67) R postcentral (60) R sup. frontal (57) R inf. parietal (47) R inf. temporal (43) R lingual (38) L olfactory (35) R amygdala (34) R olfactory (31) R mid. frontal (24) R insula (16) R Heschl (16) R Rolandic operculum (15) R hippocampus (12) Precuneus (10)
	L mid. frontal	− 18, 24, 50	3.9	837	0.008	L mid. frontal (412) L sup. frontal (163) L supplementary motor area (15)
	R lingual	16, − 32, 0	3.98	646	0.033	R lingual (92) R hippocampus (66) R thalamus (57) R cerebellum (44) Vermis (10)
**Hyper-activation in patients**						
2-back:	R angular/occipital	38, − 74, 52	4.31	901	0.005	R angular (296) R sup. occipital (223) R mid. occipital (27) R inf. parietal (18)
	L inf. parietal	− 52, − 52, 46	3.39	708	0.021	L inf. parietal (323) L angular (93) L sup. parietal (52)

*Ant.* Anterior, *Inf.* Inferior, *L* left, *mid.* Middle, *R* right, *sup.* superior.

^a^In the absence of disorder-haplotype interactions.

^b^We compared risk and protective haplotype to other haplotypes.

Individuals (patients or controls) with the risk haplotype also showed hypo-activation compared to individuals without risk haplotype, but it was substantially less extensive in the 1-back vs. baseline contrast. It only comprised the caudate, the olfactory and anterior cortices, and the rectus gyri. In the 2-back vs. baseline contrast, it was more extensive. It included the middle, superior, and inferior frontal gyri, the middle and superior temporal gyri, and to a lesser extent, the supramarginal gyrus, the caudate, the anterior cingulate cortex, and several other regions.

Finally, patients with ADHD showed hyper-activation of the inferior parietal, the angular, and the superior occipital gyri in the 2-back vs. baseline contrast compared to healthy controls.

We did not find any statistically significant effect for the other contrasts or interactions between ADHD status and *ADGRL3* haplotypes.

## Discussion

This study explored brain structural and functional differences between *ADGRL3* haplotypes, between patients with ADHD and controls, and their interactions. The main findings were the widespread strong effects of *ADGRL3* haplotypes on the brain response to work the memory task. Conversely, contrary to our hypothesis, we did not find the brain effects of the interactions between *ADGRL3* haplotypes and adult ADHD.

Specifically, individuals (patients or controls) who were homo- or heterozygous for the protective *ADGRL3* haplotype showed extensive hypo-activation compared to individuals without the protective haplotype. Surprisingly, individuals (patients or controls) who were homo- or heterozygous for the risk haplotype also showed hypo-activation compared to individuals without the risk haplotype. We must acknowledge that we do not know the functional meaning of these hypo-activations or whether they may relate or not to the protective and risk effects of these haplotypes.

Intriguingly, previous meta-analyses have shown widespread hypo-activation in patients with ADHD^17^, whereas we found them to show hyper-activation. On the other hand, hypoactivation patterns in individuals with risk ADGRL3 haplotype compared to individuals with the protective haplotype were significantly higher and more extensive in the 2-back versus baseline contrast, that is, when the difficulty of the task increased. Hypoactivation patterns have been regularly reported in studies using working memory tasks in children and adolescents with ADHD, but not consistently in adult patients with the disorder. Considering the above, a potential explanation for these opposite observations could be that the hypo-activation reported in previous ADHD studies is indeed due to the more frequency of ADGRL3 risk haplotype in the patients rather than to ADHD-related executive cognitive failures. However, other explanations are possible because activation may depend on a variety of factors. For example, individuals who do not attend the task will have little or no activation, and both the disorder and the *ADGRL3* haplotype may probably affect the attention to the task. Besides, some authors have associated activation with task accuracy but also longer reaction time^22,23^. Even methylphenidate may play a role^24^, although its involvement in working memory networks is unclear^25^. With these considerations in mind, one could hypothesize that hypo-activation in individuals with the protective haplotype and hypo-activation in patients with ADHD have different origins. For example, individuals with the protective haplotype might require weaker brain activation to attend, whereas individuals with a “tendency” to ADHD might have difficulty to activate. Under these hypotheses, individuals with a tendency to ADHD but protective haplotype would seldom develop the disorder because their lower activation requirement would cancel out their difficulty to activate. Of course, such speculations are only hypotheses. We encourage future studies to investigate them.

We failed to detect statistically significant interactions between adult ADHD and *ADGRL3* haplotypes. We found some interactions between the effects of *ADGRL3* haplotypes and disorder on brain morphometry. Still, readers should take them with caution because they did not survive the correction for multiple testing. In this regard, a recent meta-analysis has found that *ADGRL3* haplotypes confer a relevant risk in pediatric ADHD, but results were less significant in adult ADHD^9^. The meta-analysis and our study investigated different phenomena, as the former reviewed studies of the association between the haplotypes and ADHD, and the latter investigates their brain correlates. However, there is a possibility that the weaker association between *ADGRL3* haplotypes and ADHD in adults may explain why we did not found evidence of any brain effect of the interactions in our study.

Even if we failed to detect interactions between the haplotype and the disorder, we still found an impressive result: *ADGRL3* protective and risk haplotypes significantly impact the brain response to a working memory task. Interestingly, the *ADGRL3* haplotype with a larger effect was the protective one, while the risk haplotype effect was weaker.

Structural effects were not statistically significant after correction for multiple comparisons. However, this lack of statistical significance is not surprising in ADHD literature, where some studies have reported frontostriatal abnormalities that may change with age and/or treatment, while others have failed to detect them^13,15,19,20,26,27^. In agreement with previous literature, we found more robust evidence of functional brain abnormalities than structural brain abnormalities in ADHD.

This study has several limitations. First, despite our efforts to achieve a finely balanced sample, the ratio of homozygous vs. heterozygous for the protective and risk haplotypes was higher in patients with ADHD than in controls. This imbalance means that we might have potentially erroneously attributed differences between the haplotypes’ homozygotic and heterozygotic effects to the ADHD status or its interaction with haplotypes. However, this possibility seems unlikely to have had a significant consequence because our study’s main findings were indeed the haplotypes’ effects. Second, even if the global sample is moderately large for a neuroimaging study, it may still involve a limited statistical power to detect weaker effects. This little power may have affected, for example, the comparison of patients vs. controls, given the weak findings reported in previous meta-analyses^13,17^. It might also be the case for interactions between the haplotypes and the disorder. However, we were still able to detect hypo-activations that were very statistically significant, or in other words, very unlikely due to chance. Third, our sample included adults, and a recent meta-analysis reported that the effects of *ADGRL3* haplotypes depend on age^9^. Finally, we scanned the individuals in a 1.5 T device, and the functional sequence had a thickness of 7 mm with a 0.7 mm gap. We started the study with this configuration years ago. When higher resolutions were available, we decided not to change them to scan all the study participants with the same parameters. These suboptimal MRI settings may also have limited, to some degree, the statistical power of the study. Again, this potentially lower statistical power did not prevent us from detecting extensive effects of *ADGRL3* haplotypes.

To sum up, in this study, we failed to detect interactions between adult ADHD and *ADGRL3* haplotypes. Still, we found that both protective and risk of *ADGRL3* haplotypes are associated with a critical brain hypo-activation during a working memory task, a result that stresses the relevance of this gene in cognitive brain function and warrants further study.

## Materials and methods

### Participants

We recruited patients with combined type ADHD and healthy controls from Vall d’Hebron University Hospital and Benito Menni CASM. We genotyped them to obtain a sample of patients and controls balanced for age, sex, premorbid IQ, and the three *ADGRL3* haplotype groups (“risk,” “protective,” and “others,” see below). Given that haplotype frequencies differed between patients and controls, this approach involved genotyping many more individuals than those we finally included in the MRI study.

Experienced psychiatrists established the diagnosis of ADHD based on the Diagnostic and Statistical Manual of Mental Diseases, Fourth Edition, Text Revised (DSM-IV-TR)^2^ and confirmed with the Conners’ Adult ADHD Diagnostic Interview for DSM-IV (CAADID)^28,29^, the Wender Utah Rating Scale (WURS)^30^, the ADHD Rating Scale^31^, and the Conners Adult ADHD Rating Scale (CAARS)^32^. Exclusion criteria were: (a) age younger than 18 or older than 65 years, (b) left-handedness, (c) history of brain trauma, neurological disease or systemic disease with potential brain affection (e.g., congenital hypothyroidism), (d) substance use disorder (abuse/dependence) of drugs including cocaine, heroin, synthetic drugs or alcohol, (e) IQ < 70 estimated from WAIS-III vocabulary and block design subtests, and (f) comorbid major psychiatric or personality disorders. The psychiatrist assessed the latter using the Structured Clinical Interview for Axis I (SCID-I)^33^ and Axis II (SCID-II)^34^, respectively.

We recruited the healthy controls from non-medical staff, their relatives and acquaintances, and independent sources in the community. They met the same exclusion criteria as the ADHD group. We also excluded them if they: (a) took any psychotropic medication other than non-regular use of benzodiazepines or other similar drugs for insomnia, or (b) had a first-degree relative who had experienced symptoms consistent with a major psychiatric disorder and/or had received in- or outpatient psychiatric care.

The final sample of brain imaging participants was balanced for age, sex, premorbid IQ, and *ADGRL3* haplotype. We estimated premorbid IQ with the “*Test de Acentuación de Palabras*” (TAP), a test requiring pronunciation of Spanish words with accents removed^35^, analog to the National Adult Reading Test (NART)^36^. The reason to use this test is that *individuals preserve* the pronunciation of words learned before the disorder’s onset. Thus a pronunciation test may be useful to estimate the premorbid IQ^37^. We acknowledge that this estimation is more advantageous for cognitive conditions such as dementia than for ADHD. However, we considered it would still be helpful here to avoid mixing any effects of the disorder on the IQ.

The Clinical Research Ethics Committees of both Germanes Hospitalàries (for FIDMAG/Hospital Benito Menni) and Hospital Universitari Vall d’Hebron (Barcelona, Spain) approved the study. We performed all methods following the relevant guidelines and the Declaration of Helsinki and regulations, and we obtained written informed consent from all subjects before inclusion into the study.

### DNA isolation and genotyping

We isolated genomic DNA from peripheral blood lymphocytes using the salting-out procedure^38^ or saliva using the Oragene DNA Self-Collection Kit (DNA Genotek, Kanata, Ontario, Canada). DNA concentrations were determined using the Pico-Green dsDNA Quantitation Kit (Molecular Probes, Eugene, OR).

We carried out genotyping using standard PCR methods, and amplification products were tested by electrophoresis on a 1.5% agarose gel and ethidium bromide staining. We amplified the SNPs rs1868790, rs6813183, and rs12503398 using independent PCR runs. Genomic DNA was amplified for three-marker haplotype with primers Fw 5′-CTTCATTTTGTACTTTATTGAAATGTG-3′ and Rv 5′-TTCCATAGGGCAACTGATCATA-3′ for the SNP rs1868790; Fw 5′-CTCAAACCATGTTTATTCTAGACCT-3′ and Rv 5′-CAAATTATTTTCTGACCCTCTATTCTT-3′ for the SNP rs6813183; and Fw 5′-GGGTTCCAAACTTCTGATGC-3′ and Rv 5′-CCCCTCCATGAAATTCCTTT-3′ and the rs12503398. PCR reactions were carried out in a final volume of 25 µl, containing 50 ng of genomic DNA, 10 pM of each primer, 2.5 µl of PCR amplification Buffer (Invitrogen, Breda, The Netherlands), 2 mM of dNTPs, 0.5 mM MgCl, and 1U of TaqDNA polymerase (Roche). Amplification conditions consisted of an initial denaturation at 94 °C for 1 min followed by 35 cycles of denaturation at 94 °C for 1 min, annealing at 56 °C for rs1868790 and 60.3 °C for rs6813183 and rs12503398 for 1 min, and extension at 72 °C for 1 min, with a final extension step at 72 °C for 10 min. After purification of PCR products (EZNA Cycle Pure kit, OMEGA), we sequenced both strands using a Big Dye Termination system in a directly determined automated sequencing on an ABI 3130XL sequencer according to the protocol of the manufacturer. All sequencing results were analyzed using bioinformatics tools from the BioEdit Sequence Alignment Editor. *ADGRL3* haplotypes were estimated using the PHASE software^39^.

In a previous study^40^, we had found that the allelic combination with the highest association with ADHD combined type was rs1868790-rs6813183-rs12503398. Specifically, there was an over-representation of the combination T-C-A (patients: 22.9%; healthy controls: 12.8%), and thus we considered that individuals with this combination had the risk haplotype. Conversely, there was an under-representation of the combination A-G-G (patients: 13.1%; healthy controls: 17.4%), and thus we considered that individuals with this combination had the protective haplotype.

### Brain structural data

We scanned all participants in the same 1.5 T GE Signa scanner (General Electric Medical Systems, Milwaukee, WI, USA) at Sant Joan de Déu Hospital in Barcelona (Spain).

Participants underwent structural scanning with the following high-resolution T1 sequence: 180 axial slices, 1 mm slice thickness with no gap, 512 × 512 matrix size, 0.5 × 0.5 × 1 mm^3^ voxel resolution, 4 ms echo time, 2000 ms repetition time, 15° flip angle.

We visually inspected the raw structural images to check motion or other artifacts, removed the non-brain matter with the brain extraction tool (BET)^41^, and segmented the brains into gray matter and other tissues with the FMRIB software library (FSL)^42^. We had to discard twenty scans due to motion artifacts or inaccurate brain extractions or segmentations.

Gray matter segments were then normalized to MNI space with FSL as follows: (a) affine registration of the native-space gray matter images to a common stereotactic space (Montreal Neurological Institute template, MNI); (b) creation of a first template using the registered gray matter images; (c) non-linear registration of the native-space gray matter images to the first template; (d) creation of a second template using the registered gray matter images; (e) non-linear registration of the native-space gray matter images to the second template. Modulated and non-modulated images were Gaussian-smoothed with a σ = 4 mm (FWHM = 9.4 mm) kernel, which has shown to yield increased sensitivity as compared to narrower kernels^43^.

We used both modulated and non-modulated images because we have previously shown that non-linear registration can capture gross differences such as gross brain shape abnormalities, but not more subtle differences such as fine cortical thinning^43^. Thus, unmodulated images may detect the mesoscopic differences not captured by non-linear registration better. In such a case, the modulation would only introduce macroscopic noise, ultimately reducing statistical power^43^. Conversely, modulated images may better detect macroscopic differences captured by non-linear registration, as a significant part of these differences might be removed during the non-linear registration but re-introduced with modulation^44^.

### Brain functional data

We acquired the functional scans with the following gradient-echo echo-planar imaging (EPI) sequence depicting the blood oxygenation level-dependent (BOLD) contrast: 266 volumes, TR = 2000 ms, TE = 20 ms, FOV = 20, flip angle = 70°, number of axial planes = 16, thickness = 7 mm, section skip = 0.7 mm, in-plane resolution = 3 × 3 mm.

Within the scanner, participants performed a sequential-letter version of the n-back task^45^. We chose this task, which captures the active part of working memory, because impairment of working memory is one of the most robust findings in ADHD, especially in adulthood^46^. The computer presented two levels of memory load (1-back and 2-back) in a blocked design. Each block consisted of 24 letters shown every 2 s (1 s on, 1 s off), and all blocks contained five repetitions (1-back or 2-back depending on the block) located randomly within the blocks. Individuals had to indicate repetitions by pressing a button. The software presented four 1-back and four 2-back blocks in an interleaved way, with a baseline stimulus (an asterisk flashing with the same frequency as the letters) presented for 16 s between n-back blocks. The computer showed green characters in 1-back blocks and red characters in the 2-back blocks to identify which task the participant should perform. All participants first underwent a training session outside the scanner. We measured performance using the signal detection theory index of sensitivity, d’^47^. Higher values of d’ indicate a better ability to discriminate between targets and distractors.

We analyzed functional images with FEAT (fMRI Expert Analysis Tool), also included in FSL^48^. For each participant, we discarded the first ten volumes to avoid T1 saturation effects, corrected for movement using MCFLIRT^49^, brain-extracted using BET^41^, spatially smoothed using a Gaussian kernel of FWHM 5 mm, normalized to the grand-mean intensity, and filtered with a high-pass temporal Gaussian-weighted least-squares straight-line fitting (sigma = 65 s). We had to discard fourteen scans due to lack of behavioral performance (defined as negative d’ values in the 1-back and/or 2-back tasks), image artifacts, or excessive movement (defined as estimated maximum absolute movement > 3.0 mm or average movement > 0.3 mm).

We fitted within-individual general linear models, including the 1- and 2-back blocks, their temporal derivatives, and six motion parameters using FILM with local autocorrelation correction^50^. These models generated individual activation maps for the 1- and 2-back vs. baseline contrast. Individual statistical images were then co-registered to MNI space using FLIRT^49,51^.

### Statistical analysis

To assess the effects of the disorder, the impact of the *ADGRL3* haplotype and their interaction on the gray matter or BOLD response, we created a linear model with the following main binary regressors: ADHD status (patient vs. control), protective haplotype (presence vs. absence), risk haplotype (presence vs. absence), the interaction between ADHD status and protective haplotype, and the interaction between ADHD status and risk haplotype.

Beyond these regressors, we also included age, sex, and cumulative stimulant dose, given their brain structure and function relationships. With the inclusion of age, sex, and medication in the model, we both prevented their potential confounding effects (e.g., in the interactions) and decreased the residuals’ variance, thus increasing statistical power.

Structural images were analyzed using ‘threshold-free cluster enhancement’ (TFCE)^52,53^ due to its increased sensitivity compared to voxel- or cluster-based statistics^43^. We assessed statistical significance with the permutation test included in FSL and thresholded the maps using a familywise error rate < 0.05 (i.e., p corrected for multiple comparisons). With an exploratory aim, we also report results thresholded using a more liberal uncorrected p < 0.001, which has increased false-positive rate but minimizes false-negative results^54^.

We analyzed behavioral responses to the n-back task with R, and functional images were analyzed using the FMRIB’s Local Analysis of Mixed Effects (FLAME) stage 1^55,56^. Z statistic images were thresholded using clusters determined by voxel Z > 2.3 and a cluster parametric p < 0.05 corrected for multiple comparisons^57^.

## Data Availability

Data are available upon request to the Research Ethics Committee (CEI).
